# Is the Sex Difference a Clue to the Pathomechanism of Dry Eye Disease? Watch out for the NGF-TrkA-Piezo2 Signaling Axis and the Piezo2 Channelopathy

**DOI:** 10.1007/s12031-022-02015-9

**Published:** 2022-05-04

**Authors:** Balázs Sonkodi, Miklós D. Resch, Tibor Hortobágyi

**Affiliations:** 1 Department of Health Sciences and Sport Medicine, Hungarian University of Sports Science, Budapest, Hungary; 2 Department of Ophthalmology, Semmelweis University, Budapest, Hungary; 3 Institute of Pathology, Faculty of Medicine, University of Szeged, Szeged, Hungary; 4 Insitute of Psychiatry Psychology and Neuroscience, King’s College London, London, UK; 5 Center for Age-Related Medicine, SESAM, Stavanger University Hospital, Stavanger, Norway

**Keywords:** Dry eye disease, Neuropathic corneal pain, Piezo2 channelopathy, Compression axonopathy, Ganglionopathy, Glutamate, NMDA, NGF-TrkA axis

## Abstract

Dry eye disease (DED) is a multifactorial disorder with recognized pathology, but not entirely known pathomechanism. It is suggested to represent a continuum with neuropathic corneal pain with the paradox that DED is a pain-free disease in most cases, although it is regarded as a pain condition. The current paper puts into perspective that one gateway from physiology to pathophysiology could be a Piezo2 channelopathy, opening the pathway to a potentially quad-phasic non-contact injury mechanism on a multifactorial basis and with a heterogeneous clinical picture. The primary non-contact injury phase could be the pain-free microinjury of the Piezo2 ion channel at the corneal somatosensory nerve terminal. The secondary non-contact injury phase involves harsher corneal tissue damage with C-fiber contribution due to the lost or inadequate intimate cross-talk between somatosensory Piezo2 and peripheral Piezo1. The third injury phase of this non-contact injury is the neuronal sensitization process with underlying repeated re-injury of the Piezo2, leading to the proposed chronic channelopathy. Notably, sensitization may evolve in certain cases in the absence of the second injury phase. Finally, the quadric injury phase is the lingering low-grade neuroinflammation associated with aging, called inflammaging. This quadric phase could clinically initiate or augment DED, explaining why increasing age is a risk factor. We highlight the potential role of the NGF-TrkA axis as a signaling mechanism that could further promote the microinjury of the corneal Piezo2 in a stress-derived hyperexcited state. The NGF-TrkA-Piezo2 axis might explain why female sex represents a risk factor for DED.

## Background

Dry eye disease (DED) is one of the most frequent ophthalmologic disorders, affecting up to 50% of the population, and is considered a complex, multifactorial and heterogeneous disease with recognized underlying pathology but not entirely known pathomechanism (Galor et al. 2018; The epidemiology of dry eye disease 2007; Shimazaki 2018). Female sex and age are considered risk factors, and systemic cancer chemotherapy has been recently recognized as a risk factor as well (Chiang et al. 2021). Neuronal pathological alterations have been described and can be observed by in vivo confocal microscopy (Galor et al. 2018; Cruzat et al. 2017). Symptoms of DED include fluctuating and/or blurry vision and ocular dysesthesias with a “burning,” “tender” and “aching” sensation, in addition to ocular surface disturbances including decreased tear production and increased tear evaporation (Galor et al. 2018; The definition and classification of dry eye disease 2007; Kalangara et al. 2017). Earlier, DED was viewed only as a “simple” disorder of tear production and was later discussed mostly in association with autoimmune diseases (Galor et al. 2018). However, it is now considered a continuum in terms of nociceptive impairment with neuropathic corneal pain (NCP) (Dieckmann et al. 2021). The paradox of this continuum is how a pain-free eye condition in most cases can be regarded as a pain condition (Dieckmann et al. 2021). A potentially quad-phasic non-contact injury model of the Piezo2 ion channel microinjury might explain this paradox.

Furthermore, the current authors suggest that the nerve growth factor (NGF)-tropomyosin receptor kinase A (TrkA) axis signaling plays a role in promoting the development of this dose-limiting and threshold-driven Piezo2 channelopathy that may explain the sex difference in the epidemiology of DED.

## Piezo2 Ion Channel and Its Primary Microinjury

Piezo proteins are the largest membrane proteins known so far, with numerous transmembrane segments (Volkers et al. 2015). The Piezo proteins are encoded by two genes, Piezo1 and Piezo2, in humans. However, our knowledge regarding their exact topology and functions such as pore formation, mechanical force detection and gating have not been entirely explored (Volkers et al. 2015). Corneal mechanical stimuli are mainly transduced by Piezo2 channels (Coste et al. 2010; Puja et al. 2021). Nonetheless, the involvement of Piezo1 in tear production and tear evaporation should not be excluded, as these ion channels play a role in aqueous humor outflow dynamics (Zhu et al. 2021). Overexpression of Piezo1 or Piezo2 induces two kinetically distinct mechanically activated currents, where Piezo2 is responsible for the rapidly adapting ones (Coste et al. 2010).

Furthermore, Piezo2 has been identified as the principal mechanotransduction channel for proprioception in afferent somatosensory neurons (Woo et al. 2015), although these excitatory stretch-gated ion channels also mediate light touch and vibration detection (Szczot et al. 2018). It was recently hypothesized that these Piezo2 ion channels could be transiently microdamaged on a non-contact basis in an acute stress response (ASR) time window when unaccustomed or strenuous forced lengthening contractions lead to hyperexcitation, neuro-energetic depletion and eventually to mechano-energetic impairment (Sonkodi et al. 2021a, 2020).

However, strong evidence is lacking that corneal trigeminal afferents and extraocular muscle spindles contribute to the position sense of proprioception or oculomotor control (Rao and Prevosto 2013; Weir et al. 2000). Nevertheless, it is suggested that the primary afferents of extraocular muscle spindles initiate the corneal reflex (Bratzlavsky 1972). Interestingly from this perspective, Piezo2 knockout mice have diminished blink reflex capability compared with wild-type mice (Fernandez-Trillo et al. 2020). Furthermore, a recent study demonstrated that pre-pulse inhibition invoked by lower limb afferent input could contribute to postural control, where pre-pulse inhibition was measured as the percentage inhibition of the blink reflex response to electrical supraorbital nerve stimulation (Versace et al. 2019). Sonkodi et al. postulated that the overall proprioceptive system is neuro-energetically resource-limited and implicated the channelopathy of Piezo2 as a primary injury phase of an acute non-contact compression axonopathy in certain conditions, such as delayed-onset muscle soreness and non-contact anterior cruciate ligament (NC-ACL) injury (Sonkodi et al. 2021a, 2020; Sonkodi 2021). They also suggested that this Piezo2 channelopathy impairs the static encoding of the stretch reflex and could lead to a harsher secondary tissue injury and affect overall postural control (Sonkodi et al. 2021a, b, 2020; Sonkodi 2021; Sonkodi and Hortobágyi 2022). Moreover, Sonkodi et al. suggested that somatosensory afferents from the proximal tibia could contribute to the proprioceptive primary afferents of the muscle spindles and could alter the stretch reflex if it is compressively microinjured (Sonkodi et al. 2021b). Accordingly, Sonkodi and Hortobágyi proposed that corneal Piezo2-containing somatosensory terminals could contribute to the blink reflex (Sonkodi and Hortobágyi 2022), in line with the findings of Fernández-Trillo et al. (Fernandez-Trillo et al. 2020), and could also provide overall postural control in a preprogrammed way, as demonstrated by Versace et al. (Versace et al. 2019). Again, it is worth noting that the blink reflex is suggested to be evoked by the stretch of extraocular muscles (Bratzlavsky 1972).

Diclofenac, a nonsteroidal anti-inflammatory drug (NSAID), acts by a dual-action mechanism, meaning inhibition of both cyclooxygenase and lipoxygenase pathways. Diclofenac not only has a favorable effect on DOMS, suggested to be caused by Piezo2 microinjury (Sonkodi et al. 2021a), but can prevent it to some extent (Connolly et al. 2003; Cheung et al. 2003). This beneficial effect from this aspect of diclofenac is likely due to the weakening of hyperexcitation through the cyclooxygenase pathway and stabilization of membrane lipids around the Piezo2 ion channel through the lipoxygenase pathway. Notably, Piezo2 ion channels are bordered by lipids within the cell membrane (Volkers et al. 2015). The structure of these membrane lipids surrounding the Piezo2 channels could be destabilized due to the compressive stress-derived heightened lipoxygenase activity, leading to unwanted pore formation again at the Piezo2 channel, and as a result could become leaky to glutamate and Piezo currents when they should not be (Sonkodi et al. 2021a). Physiologically, Piezo channels go through inactivation in response to hyperexcitation and within homeostasis (Bewick and Banks 2021; Suchyna 2017). However, the proposed acute stress response-derived microinjury and leakiness proposed by Sonkodi et al. is beyond homeostasis (Sonkodi et al. 2021a). The possibility that the phospholipid substrate PIP2 of myotubularin-related protein-2 is harmed by the compressive stress-induced elevated lipoxygenase activity should be considered because these phospholipids are key actors in the control of the Piezo2 dependent mechanotransduction (Narayanan et al. 2018). Moreover, it could be an important underlying factor in this phenomenon that the excitatory functioning of Piezo channels depletes cholesterol locally (Buyan et al. 2020; Chong et al. 2021). Unsurprisingly, diclofenac solution and topical diclofenac are effective treatment methods for traumatic corneal abrasions and ocular pain (Szucs et al. 2000).

Corneal somatosensory polymodal nociceptors expressing Piezo2, which can be activated by mechanical stimuli, heat and chemical irritants as well (Fernandez-Trillo et al. 2020), are the prime suspects for the suggested Piezo2 channelopathy because they seem to have the capability for the cross-talk with unmyelinated C sensory fibers. The authors of this article propose that the hyperexcited and compressively injured Piezo2 channel containing terminals of somatosensory neurons on the cornea could go through terminal arbor degeneration (TAD) like mechano-energetic lesions in an acute stress response time window, as is suggested in DOMS. This TAD lesion is experienced as a side effect of platinum-analogue chemotherapy and it is dose-limiting and threshold-driven, experienced acutely and chronically as well (Sonkodi et al. 2020; Bennett et al. 2011; Vincent et al. 2016). Unsurprisingly, systemic cancer chemotherapy with agents like oxaliplatin and paclitaxel is considered a risk factor for DED (Chiang et al. 2021). Furthermore, platinum-analogue chemotherapeutic agents delay the blink reflex in cancer patients (Park et al. 2016), as Fernández-Trillo et al. showed in Piezo2 knockout mice (Fernandez-Trillo et al. 2020). Oxaliplatin only damages the static firing sensory encoding and hardy affects the dynamic ones (Vincent et al. 2016).

In addition, Oswald et al. showed that corneal epithelial wounds caused by mechanical injuries induced rapid Ca^2+^ mobilization in neuronal cells (Oswald et al. 2012). It is important to note that Piezo channels are nonselective cation channels; however, Piezo currents favor Ca^2+^ (Coste et al. 2010; Szczot et al. 2021). The current authors suggest that the mechano-energetically microinjured epithelial Piezo1 channels contribute to the abruptly released Ca^2+^ waves and hyperexcite the Piezo2 channels at the somatosensory terminals, leading to inactivation and closure of the Piezo2 channel pores.

In contrast, Oswald et al. also demonstrated that neuronal wound media invoked a more complex response in epithelial cells (Oswald et al. 2012). The current authors suggest that the mechanically micro-injured somatosensory terminal Piezo2 ion channels become leaky to Piezo currents when they should not be. Various leakage currents could divert the main logic pathway of currents. Subthreshold current is an important type of leakage current, and the current authors propose that these Piezo microinjuries induce imbalanced subthreshold currents, similar to the effects of platinum-analogue chemotherapy (Rich et al. 2020). This could explain the phenomenon in this neuronal injury scenario of Oswald et al. (Oswald et al. 2012), where late calcium waves occurred in cell clusters (Oswald et al. 2012), especially if we consider that Piezo currents favor Ca^2+^ (Szczot et al. 2021). Furthermore, the finding of Oswald et al. (Oswald et al. 2012) that *N*-methyl-d-aspartate (NMDA) antagonist preincubation of neural wound media diminishes the secondary calcium waves is in line with the theory of Sonkodi that Piezo2 channelopathy activates NMDA receptors (Sonkodi et al. 2021a, c; Sonkodi 2021). It is worth mentioning that the authors of this manuscript are only proposing Piezo2 channelopathy as the critical gateway to pathophysiology and not excluding the possible involvement of other ion channels and receptors, like TRPV1 and purinergic (P2) ones (Oswald et al. 2012), in the proposed non-contact injury mechanism.

Emerging studies provide evidence substantiating the sensory neuronal injury mechanism with central involvement. Recent findings of Luna et al. showed that unilateral corneal insult alters the activity not only of ipsilateral sensory nerves, but of the contralateral ones as well (Luna et al. 2021). The bilateral sensory involvement is not different in DOMS either, but in a non-contact manner (Marathamuthu et al. 2020; Hedayatpour et al. 2018; Courtney et al. 2020). Correspondingly, the current authors suggest that regardless of the contact or non-contact nature of corneal injury, sensory involvement could be the critical pathway.

Dieckmann et al. (Dieckmann et al. 2021) rightly addressed the aforementioned paradox of how a pain-free eye condition in most cases can be regarded as a pain condition throughout the DED and NCP continuum (Dieckmann et al. 2021). The current authors propose that the suggested mechano-energetic Piezo1 channelopathy under a stress-derived hyperexcited/overloaded environment could eventually cause microdamage to the somatosensory Piezo2 ion channels in a non-contact way and a dose-limiting and threshold-driven manner, as we can learn from chemotherapy and DOMS, due to repetitively enhanced Ca^2+^ waves. Hence, Piezo1 and Piezo2 channels could be considered two sides of the same coin, namely the Piezo system.

It is important to note that Piezo ion channels have a role in maintaining homeostasis in various tissues (Lee et al. 2014; Zeng et al. 2018; Roh et al. 2020; Woo et al. 2014; Ranade et al. 2014; Xu et al. 2021); Piezo1 channels are responsible for cell orientation (Xu et al. 2021; Li et al. 2014), regulation of osmolarity and outflow in the eye (Uchida et al. 2021), while Piezo2 is responsible for proprioception (Woo et al. 2015). However, microinjury of the somatosensory Piezo2 could impair the Piezo system or the intimate cross-talk between Piezo1 and Piezo2 channels, leading to impaired cell orientation, orthostasis and proprioception. Therefore, DED could be initiated by a chronic Piezo1 channelopathy, but could also evolve into an NCP, which is considered primarily a Piezo2 channelopathy (see Table 1), constituting the nociceptive continuum paradox. Again, it is worth noting that the suggested Piezo2 channelopathy is a critical gateway to pathophysiology through a non-contact injury mechanism, and this critical pathway and the involvement of parallel signaling pathways and multiple factors, like the impact of environmental factors and genetic predisposition (see Table 1) such as Piezo mutations, should be considered in this heterogeneous clinical picture.

**Table 1:**
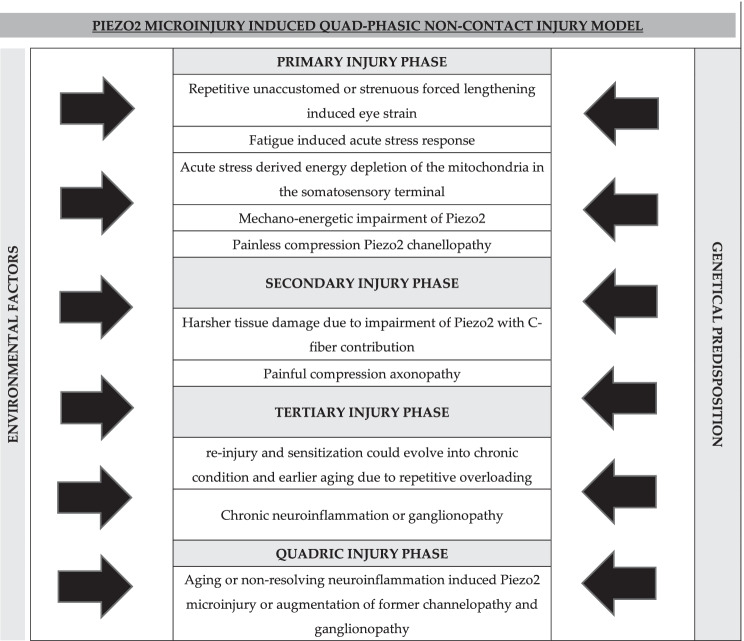
The quad-phasic non-contact injury model

## Secondary Injury Phase: Compression Axonopathy

The non-contact injury mechanism of DOMS is considered biphasic (Sonkodi et al. 2020; Hody et al. 2019; Morgan 1985), where the secondary harsher tissue damage is due to the primary injury-derived lost protection and impaired proprioception (Sonkodi et al. 2020; Hody et al. 2019), or more specifically the lost or inadequate intimate cross-talk between somatosensory Piezo2 and peripheral Piezo1. Notably, the primary Piezo2 channelopathy is suggested to be pain-free. The temporal summation of DOMS mechanical hyperalgesia, which is suggested to be an acute compression axonopathy, is provided by C-fibers that transduce the secondary phase of this harsher tissue damage (Sonkodi et al. 2020; Sufka and Price 2002; Kubo et al. 2012).

Accordingly, the current authors also propose that C-fiber contribution is needed for the evolution of NCP beyond the Piezo2 channelopathy. Indeed, the presence of loss-of-function mutations in Piezo2 impedes painful reactions to touch after skin inflammation (Szczot et al. 2018). The current authors suggest that unwanted Piezo Ca^2+^ currents due to impaired gating of Piezo2 channelopathy opens the gate to pain sensation, but C-fiber contribution is essential for transmitting nociceptive information in NCP (see Table 1). Furthermore, Perini et al. demonstrated that the lower the peripheral C-fiber density, the lower the pain intensity (Perini et al. 2016).

## Tertiary Injury Phase: Ganglionopathy

Importantly, in the presence of loss-of-function mutations in Piezo2, there is not only the absence of pain, but the development of sensitization is missing as well (Szczot et al. 2018). The current authors suggest that these Piezo2 mutations impede the Piezo part of the early and late calcium waves, as shown by Oswald et al. (Oswald et al. 2012). Moreover, it was demonstrated on bovine cornea that these calcium waves seem to play an important role in wound healing (Chifflet et al. 2012). Accordingly, the authors of this manuscript propose that the chronic somatosensory Piezo2 channelopathy or the permanent unwanted leakiness of these ion channels could also be implicated in the sensitization process, which could be seen as low-grade neuroinflammation, and part of the wound healing is kept alive permanently instead of transiently. Correspondingly, there could be two pathways for sensitization, namely non-injured Piezo2 with enhanced and imbalanced Ca^2+^ waves due to Piezo1 microinjury and microinjured or leaky Piezo2 with enhanced calcium waves.

Recent findings of Tei et al. (Tei et al. 2022) demonstrated that the α_2_δ-1 subunit of the pore-forming α_2_δ voltage-gated Ca^2+^ channels was significantly increased in the dorsal root ganglion (DRG), leading to increased transport to the central nerve terminals of these pseudounipolar trigeminal neurons, constituting a critical mechanism in sensitization (Tei et al. 2022). The authors of this manuscript suggest that one important consequence of the leaky Piezo2 channelopathy-derived enhanced calcium waves on the peripheral terminal of the same pseudounipolar somatosensory neurons is the upregulation of α_2_δ-1 protein in the DRG on the injured side, thereby opening the pathway to ganglionopathy. Furthermore, these enhanced and imbalanced Ca^2+^ waves entering the central nervous system (CNS), with concomitant NMDA receptor activation, could lead to the activation of microglia and opening of the pathway to neuropathic pain (Sonkodi 2021; Tei et al. 2022; Tsuda 2016). (Tei et al. 2022) also showed that DED hypersensitivity and hyperalgesia progressively decreased the number of inhibitory interneurons in the trigeminal nucleus of the CNS in a degeneration-related manner (Tei et al. 2022). In their study, microglia activation peaked at 3 days, while astrocyte activation began only at 2 weeks and was sustained thereafter. Notably, persistent activation of astrocytes is known to play a critical role in the maintenance of neuropathic pain symptoms (Tei et al. 2022). Also importantly, Sonkodi et al. (Sonkodi et al. 2021a) proposed that the time interval of the non-contact primary injury phase (transient Piezo2 channelopathy) shown in the absence of secondary injury or acute axonopathy is up to 3 days (Sonkodi et al. 2021a).

The hypothesis and findings of Tei et al. (Tei et al. 2022) also correlate with the hypothesis of Sonkodi et al. (Sonkodi et al. 2020), namely that the pathological hyperactivation of primary sensory neuron terminals could lead to the activation of second-order neurons, leading to hypersensitivity and hyperalgesia (Sonkodi et al. 2020; Tei et al. 2022). The current authors suggest that the enhanced and imbalanced Ca^2+^ waves due to Piezo2 channelopathy that is suggested to lead to the upregulation of α_2_δ-1 protein, eventually decreases the conduction velocity of primary sensory neurons, as Sonkodi et al. suggested (Sonkodi et al. 2020), leading to the switch of involved transduction to second-order neurons.

We can learn from DOMS that non-contact injuries have longitudinal dimensions, like the repeated bout effect in the case of DOMS (Nosaka et al. 2001). Proprioception is related to memory and learning; hence the microinjury of these somatosensory terminal Piezo2 channels is proposed to open memory pathways in the CNS primarily due to the activation of NMDA receptors (Sonkodi 2021). Oswald et al. found that NMDA antagonist preincubation of neural wound media diminished the secondary or late Ca^2+^ waves (Oswald et al. 2012). Sonkodi et al. suggested that this tertiary non-contact injury phase is the consequence of repeated transient re-injury of Piezo2 channels or chronic Piezo2 channelopathies with memory dimensions (Sonkodi et al. 2021a, b). Furthermore, they suggested that the tertiary injury phase could evolve even in the absence of the secondary injury phase (Sonkodi et al. 2021a). Accordingly, the corneal somatosensory nerve endings work alongside the tear film; therefore, overloading them could lead to vulnerability to repeated damage under conditions of inflammation or repetitive environmental injury (Galor et al. 2018), constituting the tertiary phase of DED and NCP.

We can further learn from the tertiary injury phase of NC-ACL injury when full proprioceptive neuron terminal regeneration is absent. In addition to the primary role of Piezo2 channels in proprioception, ASIC3 acid-sensing ion channels are secondarily involved in proprioceptive mechanotransduction (Woo et al. 2015; Lin et al. 2016). ASIC3 plays a crucial role in secondary hyperalgesia of joint inflammation in animal models, but not in primary hyperalgesia (Niibori et al. 2020; Ikeuchi et al. 2008). The ASIC3 acid channels were found to undergo gradual upregulation in the DRG primary afferent neurons of knee joints in osteoarthritic rats, and immune cells were activated in the neural tissue as a consequence of this secondary hyperalgesia and the progression of osteoarthritis (Niibori et al. 2020). Furthermore, inflammatory signaling and genetic reprogramming were found to sensitize Piezo1 channels in the secondary injured chondrocytes on the periphery as a pathogenic feed-forward mechanism of osteoarthritis (Lee et al. 2021a). Correspondingly, DED, which is a multifactorial corneal disease accompanied by neurosensory abnormalities and even NCP, upregulates TRPV1, TRPA1, ASIC1 and ASIC3 mitochondrial RNA (mRNA) in the ophthalmic branch of the trigeminal ganglion (see Table 1) (Fakih et al. 2021). Activation of the ASIC3 channels in the brain modifies the acid-evoked currents that lead to fear conditioning (Vralsted et al. 2011). A high percentage of patients with DED experience fear and pain sensitivity to light (photophobia), a phenomenon suggestive of central sensitization with the involvement of ASIC3 channels (Galor et al. 2016; Ortega-Ramirez et al. 2017). It is important to note that even blind patients can experience photophobia (Galor et al. 2016). Patients suffering from DED are more likely to experience severe psychological stress, anxiety and depression (Na et al. 2015), and similarly, patients with depression, stress and anxiety are more susceptible to DED (Yilmaz et al. 2015). Notably, it was shown in ASIC3-knockout mice that ASICs contribute to depression and anxiety (Chu and Xiong 2012). Moreover, ASIC3 channels could be responsible for longitudinal memory formation (Vralsted et al. 2011) once the primary Piezo2 channel microdamage activates NMDA receptors, thereby opening the memory pathways, including immune memory (Sonkodi et al. 2021a, b; Sonkodi 2021). The persistent overloaded cross-modulation of Piezo and ASIC3 channels is likely under these chronically impaired circumstances. It is important to emphasize again that the possible involvement and cross-modulation of other ion channels, such as TRPV1, cannot be excluded.

Pituitary adenylate cyclase-activating polypeptide (PACAP) is capable of suppressing DED symptoms by stimulating tear secretion (Nakamachi et al. 2016). Nakamachi et al. demonstrated that PACAP-derived tear secretion involves the adenylyl cyclase/cyclic adenylyl cyclase monophosphate/protein kinase A (AC/cAMP/PKA) cascade in enhancing the translocation of the water channel protein aquaporin 5 (AQP5) from the cytosol to the membrane of the acinar cells, thus increasing water permeability (Nakamachi et al. 2016). PACAP also plays a crucial role in regulating the stress response, even at the cellular level, in addition to preserving neuronal energy homeostasis (Rudecki and Gray 2016; Stroth et al. 2011). This could have relevance since the primary Piezo microlesion of DED is suggested to be a stress-induced mechano-energetic one. Borbiro and Rohacs showed that cAMP is capable of enhancing the activity of Piezo2 (Borbiro and Rohacs 2017); therefore, cAMP shortfall in cell membranes as a consequence of chronic stress-derived relative PACAP deficiency could also have a role in Piezo microdamage.

Both innate and adaptive immune responses are involved in the pathogenesis of DED. The activation of pattern recognition receptors (PRRs) is critically involved in this sterile inflammatory process by reacting to the release of endogenous stimuli (Lambert et al. 2020; Kigerl et al. 2014). These endogenous molecules are known as damage-associated molecular patterns (DAMPs) (Kigerl et al. 2014; Tang et al. 2012). DAMPs are released into the cytoplasm as a result of CNS injury, and the chronic activation of these receptors can lead to inflammatory diseases (Kigerl et al. 2014). Heat shock proteins and high-mobility group box 1 (HMGB1) are examples of DAMPs on the ocular surface, and indeed higher levels of HMGB1 DAMPs have been found in DED patients (Alven et al. 2021). A link has been shown between ion channel expression and the activated innate immune system and inflammatory response in the pathogenesis of several diseases (Han and Yi 2014).

We can learn from an overlapping pain condition, called post-orgasmic illness syndrome (POIS), that spermidine depletion-induced opioid-like withdrawal could be an additional stress source during stress-derived fatiguing repetitive forced lengthening contractions (Sonkodi et al. 2021a). Accordingly, one important symptom of POIS is transient burning in the eyes after repeated bouts (Waldinger 2016). Joubert et al. (Joubert et al. 2020) recently demonstrated in mice that corneal sensitization is modulated by endogenous opioid peptides via binding to μ, δ and κ opioid receptors in the CNS (Joubert et al. 2020; Giannaccare et al. 2021). Reaux-Le Goazigo et al. (Reaux-Le Goazigo et al. 2019) also showed in mouse models that topical administration of endogenous opioid peptide-degrading enzyme inhibitor seemed to significantly alleviate corneal pain and inflammation (Giannaccare et al. 2021; Reaux-Le Goazigo et al. 2019). Furthermore, it was found that Leu-enkephalin (derived from the same aforementioned endogenous opioid peptide precursors) could also facilitate wound repair through the modulation of matrix metalloproteases (Yang et al. 2016), thereby decelerating the extracellular matrix degradation process (Giannaccare et al. 2021). Extracellular matrix is an important medium, as this is where the locus of pain sensation by C-fibers correlates more strongly with the harsher tissue damage experienced in the secondary non-contact injury phase. However, the locus of primary Piezo2 microdamage could be different, as in DOMS, where it is suggested to be in the muscle spindle. It is important to note that topical administration of spermidine on DED is protective as well (Lee et al. 2021b).

Neuropathic pain is an important hallmark of NCP and has been considered as a lesion or disease of the somatosensory nervous system (Galor et al. 2018). In some cases, the underlying pathology with trauma to the nerves is better known, as in diabetic neuropathy, post-herpetic neuralgia and some cases of chronic postoperative pain (Galor et al. 2018). In other cases, the underlying pathophysiology remains unknown, such as in cases of temporomandibular joint disorders, chronic fatigue, irritable bowel syndrome, interstitial cystitis, vulvodynia, burning mouth and fibromyalgia, in addition to NCP (Galor et al. 2018), but even POIS in a later stage could be counted here as well (Sonkodi et al. 2021a). Nonetheless, a common hallmark of these conditions is that they often co-exist in an overlapping fashion and are linked by neuropathic mechanisms, i.e., peripheral and/or central sensitization (Galor et al. 2018). The current authors attribute this overlapping and interlinked feature of these conditions principally to the tertiary non-contact injury phase of underlying Piezo2 channelopathy of somatosensory terminals or the microinjury of the Piezo system, especially under stress-derived fatiguing repetitive forced lengthening contractions when neuro-energetic resources are scarce due to the phenomenon that these resources are limited (Sonkodi 2021).

Additional discussion of peripheral and central sensitization of corneal pain and their progressiveness is not the subject of this manuscript, only the aforementioned critical pathways. However, comprehensive reviews about sensitization are presented by Galor et al. (Galor et al. 2018) and Puja et al. (Puja et al. 2021).

## The Quadric Injury Phase: Aging-associated Neuroinflammation

Again it is worth noting that increasing age has been considered a risk factor for DED (Chiang et al. 2021), and therefore it seems that aging-associated low-grade neuroinflammation could initiate or augment the low-grade neuroinflammatory consequence of corneal somatosensory Piezo2 channel microinjury, revealing a clinical picture with higher prevalence. As aging increases lipid peroxidation (Spiteller 2001), this could further increase the aforementioned membrane lipid depletion around the Piezo channels. Moreover, lipid peroxidation in mitochondria also contributes significantly to age-related pathologies (Ademowo et al. 2017). Therefore, aging-associated neuroinflammation, or inflammaging, could be called the quadric phase of this non-contact injury model (see Table 1). It is no surprise that ASIC3 and other acid-sensing ion channels, implicated earlier in cornea sensitization, seem to be a promising pharmacological target for neurodegeneration and neuroinflammation (Ortega-Ramirez et al. 2017). Nevertheless, the aging mechanism and associated inflammaging are outside the scope of this paper. It is important to mention that PACAP deficiency imitates aging-associated pathophysiology and promotes earlier aging (Reglodi et al. 2018), as Nakamachi et al. demonstrated in the cornea of PACAP-null animals (Nakamachi et al. 2016).

## Sex Difference: Female Sex As a Risk Factor

Female sex is considered a risk factor for DED (The epidemiology of dry eye disease 2007); however, the significant difference in prevalence compared with men evolves only with age (Stapleton et al. 2017). Sonkodi et al. attributed the sex difference in the occurrence of NC-ACL injury to the NGF-TrkA signaling axis on proprioceptive sensory neuron terminals, which is the suggested locus of the primary microinjury of this dichotomous non-contact injury mechanism (Sonkodi et al. 2021b). The current authors suggest that the excessively elevated PGE2 levels might explain the phenomenon that females are more prone to DED and presumably to other mucosal dryness syndromes, as was demonstrated by Gebri et al. (Gebri et al. 2020). In the pre-ovulatory phase of the menstrual cycle, when a marked elevation of estrogen is due to luteinizing hormone (LH) (Sonkodi et al. 2021b), LH also stimulates the NGF-TrkA axis through interleukin-1β in the ovarian cells and promotes TrkA and NGF gene expression and PGE2 release (Dissen et al. 1996). This mechanism could further elevate PGE2 in excess of the levels generated by hyperexcited corneal cells due to mechanical stress.

Later, Sonkodi et al. even implicated the Piezo2 channelopathy in these proprioceptive terminal microinjuries leading to non-contact injuries (Sonkodi et al. 2021a, c; Sonkodi and Hortobágyi 2022). Indeed, recent findings of Nencini et al. showed that the NGF-TrkA-Piezo2 signaling axis has an influence on Aδ sensory afferent neurons in noxious mechanical stimulation, and that Piezo2 is also a player in the sensitization of these neurons to mechanical stimulation (Nencini et al. 2021). The current authors propose that the microinjury of Piezo2 ion channels at the Aδ sensory terminals in the cornea is the gateway to pathophysiology towards DED/NCP, and the NGF-TrkA axis signaling on these somatosensory neurons promotes this microinjury mechanism. Additionally, the NGF-TrkA-Piezo2 axis could be the critical signaling explaining the sex difference in DED.

Importantly, Nakamachi et al. also found that PACAP-null female animals were more affected than males (Nakamachi et al. 2016). The current authors suggest that the “energetic” part of the mechano-energetic Piezo microdamage, namely stress-derived inadequate cAMP mobilization towards Piezo2 functionality through the AC/cAMP/PKA cascade in PACAP-null animals, is independent of sex regardless of estrogen’s ability to increase cAMP. On the contrary, the “mechano” part of the stress-associated microlesion involves the NGF-TrkA-Piezo2 signaling machinery, and it could account for the sex difference. Moreover, estrogen-containing hormone replacement therapy (HRT) might also play a role in sex difference presentation. Estrogen by itself as HRT increased the risk of DED by approximately 70%, and in combination with progesterone increased it by approximately 30% (Schaumberg et al. 2001). However, the authors suggest that this estrogen effect is rather due to lipid catabolism in addition to aging-associated lipid peroxidation, leading to enhanced membrane lipid depletion around the Piezo2 ion channels and not through the cAMP signaling pathway. The negative influence of estrogen on lipid synthesis should also not be excluded as a contributing factor of DED prior to menopause.

## Conclusions

DED is the most common ophthalmologic disorder, with a substantial economic burden and impaired quality of life. This paper proposes a critical pathophysiological role of corneal somatosensory Piezo2 channelopathy at the nerve terminals in the development of DED. This microinjury could open a gateway to a potential quad-phasic non-contact injury mechanism on a multifactorial basis and with a heterogeneous clinical picture. Furthermore, the NGF-TrkA-Piezo2 axis may be the critical signaling explaining the sex difference with female predisposition in DED.
